# “My Cross-Border PhD Journey”: A Qualitative Study on the Educational and Life Challenges of Mainland Chinese PhD Students in Hong Kong

**DOI:** 10.3390/ijerph20126078

**Published:** 2023-06-07

**Authors:** Jing Jia, Nelson C. Y. Yeung

**Affiliations:** 1 The Jockey Club School of Public Health and Primary Care, The Chinese University of Hong Kong, Hong Kong SAR 999077, China; 2 Faculty of Education, The Chinese University of Hong Kong, Hong Kong SAR 999077, China

**Keywords:** Chinese PhD student, stressor, Hong Kong, qualitative study, focus-group interview, educational and life transitions (ELT)

## Abstract

PhD students’ poor mental health has been increasingly concerning. However, challenges among PhD students studying aboard are understudied. The Educational and Life Transitions (ELT) model postulates that international PhD students are subject to both academic and acculturative stressors; however, relevant research is limited in the Chinese context. We examined mainland Chinese PhD students’ study and living experiences in Hong Kong using a qualitative approach. Through purposive sampling, 37 mainland Chinese PhD students in different disciplines from public-funded universities in Hong Kong were recruited to participate in online focus group interviews (December 2020–February 2021). The interviews were analyzed using the framework analysis method. Ten themes of academic/acculturative stressors were identified. The academic stressors included: (1) high expectations from the supervisors; (2) emphasis on self-discipline as PhD students; (3) peer comparison in academia; (4) difficulties shifting research directions/academic disciplines; (5) uncertainties about future career. The acculturative stressors included: (1) differences in the political environment; (2) language barriers; (3) difficulties living in Hong Kong; (4) limited social interaction with others; (5) local people’s discriminatory behaviors. This study sheds light on the stressors experienced by mainland Chinese PhD students in Hong Kong. To better address those students’ academic and acculturative stressors, cross-cultural training and additional support from supervisors/the university could be provided.

## 1. Introduction

At present, there is a mental health crisis in academia [1]. According to the latest Nature’s global survey of graduate students in 2022, which included 3253 graduates from six continents (87% came from Europe, North America and Asia), over one-third of PhD respondents report that they have received help for anxiety or depression caused by their study [2]. This rate is far higher than the 12% found in Nature’s global survey in 2017 [3], which calls for urgent attention to the mental health of PhD students.

“Doctoral researchers are a key part of universities, they’re future thinkers, innovators and leaders that are vital in building and sustaining our economy”, as said by Professor Jeremy E. Niven, Dean of the Doctoral School at the University of Sussex, who led a nationwide study of the mental health of UK doctoral researchers [4]. As a result of this importance, concerns for PhD students’ well-being have been growing in academic research. Studies from Australia, Belgium and the U.S. showed that PhD students experienced a greater incidence of mental illness than the general population [5,6,7]; their mental health risk was also higher than that of master’s students in the higher education field [8]. Meanwhile, evidence from three systematic reviews suggested that PhD students studying in their home countries (e.g., U.S., U.K., Canada, Australia, Finland, Netherlands, China, Malaysia) are at risk of poor psychological well-being [8,9,10]. We could expect that PhD students studying overseas might suffer more due to the exposure to other aspects of stressors as sojourners.

According to the Educational and Life Transitions (ELT) model [11], international doctoral students not only need to go through transitions in a new educational system, but also face challenges living in a new society. The ELT model clearly differentiates international university students from other cross-cultural sojourners, such as business expatriates and short-term exchange students [12]. It incorporates a holistic look into the analysis of adaptation to daily and academic life among international university students [13].

Although the ELT model is designed for international doctoral students, relevant research on the PhD population is scarce. In addition, most of the previous empirical research only investigated one type of transition in the ELT model, while the double transitions were not valued equally in these studies. Adopting the transition concept pointed out by the ELT model, Pho and Schartner [14] used a mixed-methods approach to explore the social contact patterns of 101 international students undertaking taught postgraduate degrees in the U.K., and examined its relationship with academic achievement and adaptation. The findings suggested that social contact is an important contributory factor for academic adaptation and that more contact with other non-co-national international students provided important emotional and academic support. Najjar [15] conducted interviews among 34 international doctoral students from a midwestern research institution in the U.S. to investigate their advising relationships and adaptation experiences. The findings revealed specific obstacles related to language difficulties and interaction within/outside the academic environment, immigration or visas and family and financial concerns (personal incomes and academic financing). Byrne, et al. [16] adopted the focus group interview design among 31 international medical students in an Irish university to explore their challenges in social adaptation and interactions. The findings revealed the positive and negative aspects of daily-life transition and highlighted that making friends, as well as cross-cultural communication, were the main challenges facing international medical students. McGarvey, et al. [17] conducted focus group interviews among 31 international medical students in their senior clinical years in a medical university located in Ireland to explore the impact of the participants’ cultural backgrounds and language on their transition as international medical students to the clinical years of their training. The findings pointed out the daily life challenges (e.g., cultural integration and poor transportation) and academic challenges (e.g., language, practice religious belief) among these senior international medical students.

PhD students are subject to a spectrum of academic stressors throughout the PhD journey; for example, time pressure for work-life balance and worries about delayed graduation, uncertainty of the PhD process (i.e., finding a clear research direction independently, the ambiguity of completing a doctorate, uncertain career prospects), financial concerns and their supervisory relationship [18,19,20]. Meanwhile, international students often have many difficulties when moving to a foreign country to study, such as language barriers, loneliness, discrimination and practical problems associated with changing environments [21]. Thus, they may experience acculturative stress (i.e., psychological stress resulting from the acculturation process [22]) and may have social, psychological and behavioral changes [23].

The intensity of acculturative stress is strongly influenced by the similarities or dissimilarities between the host culture and that of the new entrants. The more differences between the host culture and the newcomer’s native cultures, the more acculturative stress will be experienced [24]. Therefore, it is plain to see that Chinese international students in America, Europe, Oceania or other western countries are more likely to suffer from culture shock or adaptative problems [15,25,26]. However, in some Asian countries (e.g., Korea, Japan, Singapore, Malaysia), although there may be more similarities with the Chinese culture, Chinese international students also report having experienced acculturative stress and adjustment challenges [27,28,29,30,31]. Noteworthily, research has shown that people moving to another region within the same country (i.e., internal migration) may also encounter ‘culture shock’, parallel to international migration [32]. In the Chinese context, emerging studies have examined internal migration (mainly rural-to-urban) and health among children, adolescents [33,34] and migrant workers [35,36] in China; however, students who migrate for pursuing higher education are usually ignored.

Due to special historical reasons (i.e., Hong Kong was a colony and later a dependent territory of the British Empire from 1841 to 1997), Hong Kong has been influenced by both Eastern culture and Western culture. This unique cultural environment attracts many mainland students coming to Hong Kong for pursuing higher education. The data suggest that research postgraduate (RPg) students recruited from mainland China occupied 67.3% of the total RPg students (*n* = 7602) in Hong Kong in 2017–2018, which is a 15.59% increase from 2007–2008 [37]. These mainland PhD students constitute the largest group of non-local students in Hong Kong. However, even though Hong Kong is a place with higher geographical proximity and cultural similarity with mainland China than other countries, it is still common for these mainland Chinese students to encounter difficulties while studying and living in Hong Kong. Some of them ‘feel lonely and stressed’, ‘encounter language barriers’, ‘feel discrimination’, ‘experience cultural differences’ and ‘feel difficult to integrate into local life’ [38,39,40]. Specifically, mainland students meet language barriers when adapting themselves to a bi-lingual environment (English for academic purposes and Cantonese for social interaction [41]) when studying in Hong Kong [38]. Cultural differences refer to the subtle difference between a subculture (Hong Kong) and its mother culture (Mainland China), such as the social norm, living style [40], as well as differences in the academic cultural environments [42]. Mainland students also have limited social network/interaction, and even suffer from social isolation or social exclusion [43,44]. Although prior studies have focused on mainland Chinese undergraduate or master’s students in Hong Kong, studies specifically on PhD students are limited; we can still expect that mainland Chinese PhD students in Hong Kong may also experience difficult times in their educational and life transitions.

Studies on the dual levels of challenges (academic and acculturative) among mainland Chinese PhD students’ adaptation in the intranational settings are limited [45]. Most of the research investigating Chinese international PhD students has focused on academic adjustment and was conducted in a particular foreign country outside of China. For instance, Hu and Zhao [46] explored the interactions between Chinese doctoral students and their supervisors at Dutch universities, using the interviews approach, to reveal the challenges in fostering independence in such an intercultural context. Wang and Räihä [47] used the case-study approach to explore how Chinese doctoral students integrate into the Finnish learning environment. Zhang [48] explored the transition experiences of international Chinese doctoral students at a U.S. research university. Furthermore, no local study to date has specifically examined the educational and life transitions among mainland Chinese PhD students in the context of within-country migration. Therefore, to fill in this research gap, and guided by the ELT model, this study aimed to examine mainland Chinese PhD students’ study and living experiences in Hong Kong using a qualitative approach.

## 2. Methods

This study used focus group interviews for obtaining qualitative data from the participants. Compared to individual interviews, focus group interviews can obtain information about the similarities and differences in people’s perspectives in a group setting, as well as generating large amounts of data in a relatively short time [49]. Focus group interviews have been commonly used in qualitative/mixed-method studies exploring international students’ acculturative and academic experiences [40,50,51,52].

### 2.1. Recruitment

Eligible participants of this qualitative study were: (1) full-time mainland Chinese PhD students enrolled in one of eight publicly funded universities in Hong Kong; (2) aged ≥18; (3) holding a student visa issued by the Hong Kong immigration department; (4) having been studying in Hong Kong for at least 6 months (to ensure they had some experience adjusting into the local society and academic studies); (5) Mandarin-speaking.

Prospective participants were recruited with purposive sampling through multiple channels. For example, the electronic study announcement of this study was posted through the postgraduate students’ mass e-mail system. Similar to a previous study [53], we collaborated with the Chinese Students and Scholars Association (CSSA) of the eight publicly-funded universities in Hong Kong to distribute the study flyer on their WeChat official accounts of those associations and alumni’s WeChat groups. Regardless of the recruitment channels, the study flyer described the purpose, eligibility criteria and procedure. A web link and QR code, which was generated on an online survey platform (Tencent Questionnaire), were embedded at the end of the study flyer. The study was conducted from December 2020 to February 2021.

### 2.2. Preparation of Interview Outline

A semi-structured, open-ended interview protocol was prepared for this study. Guided by the ELT Model [11], acculturation experience and academic experience were derived as priori themes in the interview outline. The interview questions were developed with reference to other relevant studies on mainland students’ adjustment in Hong Kong [40,54] and international doctoral students’ academic and adaptation experiences [15,55].

The interview protocol was pilot tested among 6 postgraduate students from mainland China who major in public health to ensure the questions were relevant to the target population’s experiences. After obtaining feedback from the pilot interviews, the questions were reviewed and discussed again among the authors for the finalization of the interview protocol. The interview questions are listed in Supplementary Materials Table S1.

Interested participants were invited to register for the study by contacting the first author individually or filling in the registration form attached to the study flyer. After checking eligibility and obtaining online informed consent, 6–8 interviewees were assigned to each group based on their characteristics (e.g., institutions, academic disciplines, stage in the PhD journey) and preferred interview time. Each focus group interview was scheduled on the weekend.

Participants were recommended to participate in the interviews in a quiet room with proper lighting and to check the stability of their Internet network. A reminder e-mail with detailed interview information (e.g., login information for the online focus groups) was sent to participants.

Considering the COVID-19 pandemic situation and social distancing measures in Hong Kong, the online focus group interviews were conducted and recorded via Zoom. The first author served as the moderator, conducting the interviews and facilitating the focus group process. As suggested by Millward [56], the direct involvement of the researcher is useful because of their rigor in methodology and sensitivity to the issues. The focus group interviews were conducted in Putonghua, the native language of the target population and the focus group moderator. Participants were asked to turn on their cameras until the end and were reminded that the interviews were audio-recorded. The participants could use nicknames/alias during the interview. Each interview lasted around 90 min. Participants who finished the focus group interviews were given RMB 50 through WeChat pay or Alipay as compensation for their time. This qualitative study has been approved by the Survey and Behavioral Research Ethics Committee at the authors’ institution (Reference No. SBRE-19-575).

### 2.3. Data Management and Analysis

The audio recordings of the interviews were translated using Notta (a professional audio-to-text transcribing software). The interviews were then reviewed by the first author and double-checked by another trained coder (a mainland PhD student from Social Science, with a linguistics degree). Microsoft Word (embedded with DocTools, with a function to extract and export text from Word to other platforms) and Microsoft Excel were used for extracting and coding the qualitative data. Specifically, the audio files were transcribed verbatim to text, the text was transferred from Word to Excel and coded in Excel, then the coded interviews were sorted by the relevance of the codes and the quotes and references were transferred from Excel to Word; then, the texts were sorted into a logical structure (i.e., priori themes) based on the coding and, finally, analyzed by two coders individually [57]. Framework analysis was used to guide the coding and analysis process of the qualitative data, which consists of five key stages: familiarization, identifying a thematic framework, indexing, charting, mapping and interpretation [58]. Guided by the ELT model, academic stressors and acculturative stressors were classified as “a priori” themes [59] before identifying the matric framework.

To guarantee the trustworthiness of the data and reduce bias, two coders (the first author and the trained student) coded the transcripts independently [60,61]. The two coders reviewed the codes and classified them into themes and subthemes. When discrepancies in the codes emerged, the coders discussed and resolved them to reach a consensus. Inter-Rater Reliability (IRR) was used to measure the extent to which the different researchers agreed on how to code the same content [62]. Researchers are suggested to calculate both the percent agreement and Cohen’s kappa to reduce the limitations of both IRR measurements [63]. Specifically, percent agreement = number of agreements/(number of agreements + disagreements) [64] and an 80% agreement between coders on 95% of the codes is sufficient agreement among multiple coders. Cohen’s kappa = (actual observed agreement − chance agreement)/(1 − chance agreement) and 0.81–1.00 represents perfect agreement between the raters [63]. After the calculation, the percent agreement was 95.1% among all the codes and the Cohen’s kappa was 0.9; both indicated a very good agreement between the two coders in this study.

### 2.4. Sample Size Planning

Factors determining the sample size in qualitative studies include the topic, data quality and study design. The number of participants to be recruited was determined by data saturation [65]. Based on previous studies on the acculturation experiences of mainland Chinese students in Hong Kong [38,40], data saturation is generally achievable with a sample size of 32. For each group interview, 6–8 participants are recommended to show the greatest potential of the group discussion and information saturation [66]. Among 42 prospective participants expressing interest in this study, 37 were eligible.

## 3. Results

### 3.1. Participants’ Characteristics

Among the 37 mainland Chinese PhD students, nearly half (48.65%) were female, over 70% (70.27%) were aged 23–27 and over half (62.16%) were year 2 and year 3 PhD students. In regard to their majors, the same proportion of students (24.32% majored in Sciences and Engineering, followed by Social Science (18.92%) and Medicine (16.22%). Over 40% (40.54%) of the students have studied in Hong Kong for more than 3 years, then 2–3 years (24.32%), 0.5–1 (18.92%), with the least being 1–2 (16.22%) (Table 1).

### 3.2. Themes

Guided by the ELT model, ten subthemes from the two major aspects of stressors (academic stressors/acculturative stressors) were derived from the focus group interviews. A summary of the themes and subthemes is presented in Supplementary Materials Table S2 and Figure S1.

#### 3.2.1. Theme 1: Academic Stressors

Most of the participants believed that their academic journey was stressful. Their stressors consisted of high expectations from the supervisors, emphasis on self-discipline as PhD students, peer comparison in academia, difficulties shifting research directions/academic disciplines and uncertainties about their future career (Table 2). The participants’ academic stressors seemed to vary across their disciplines; with or without relevant research experience, the quality of the supervisory relationship also makes a difference.

1. High expectations from the supervisors

The participants agreed that the supervisor plays a vital role in the academic journey, and many reflected on their tendency to follow the supervisor’s guidance and expectations. High expectations from supervisors usually pose a challenge to the students. In addition to the program requirement, supervisors usually have requirements about the quantity and quality of publications, especially for students from the Science faculty. Students from the Arts or Social Science faculties experienced less pressure to publish, which might reflect some differences in PhD education across disciplines.

*Patrick: “My supervisor likes to do pioneer work and make outstanding contributions to one field; instead of repeating what someone else has already done, he hopes us do that kind of challenging thing, but it will be quite difficult at the beginning for us students.”*

*Kaylee: “My supervisor has a very clear expectation on the students’ publication, she asks me to publish five to six papers, expects me not just to meet the minimum requirements (set by the program), she also set a higher standard.”*

*However, some students believed that their supervisors did not provide them with adequate guidance. For example, some supervisors only showed little concern or provided little guidance (due to lacking relevant expertise) to students’ research, which increased students’ difficulties to progress, or even delayed their graduation.*

*Xing: “My research requires me to do experiments, my supervisor is good at doing modeling but completely not skilled in doing experiments… I found it quite difficult, which was equivalent to starting from zero, roughly.”*

*CY: “There is no guidance on my thesis project… delay graduation is a common phenomenon in our faculty, no student guided by our supervisor could graduate on time.”*

2. Emphasis on self-discipline as PhD students

Many participants found that conducting research as PhD students strongly emphasizes self-discipline and independence. PhD education is regarded as a journey that requires a high level of independence and skills to manage one’s time and research-related problems. Students need to make good use of the limited time and deal with problems they encountered to graduate in a reasonable timeframe.

*Stacey: “PhD study requires us to have a strong ability to be self-managed.”*

*John: “Everyone is very independent in their work, and there is no one to discuss with except the supervisor. Moreover, when you have problems, you cannot always ask the supervisor to answer you. And then you may get stuck in one thing that you don’t know what to do, that’s the hardest part.”*

*Panda: “Unlike previous study or work, I have a clear task list and timeline to finish every day; I feel more stressed in the doctoral study is that I often don’t know how long to finish one task (I often can’t comply with my schedule). Also, PhD study requires me to have a higher level of time management or the ability of self-control.”*

3. Peer comparison in academia

Peer comparison in academia was commonly mentioned by the participants. Those students might compare their performances with previous college classmates, PhD classmates enrolled in the same year, PhD students guided by the same supervisor and other PhD graduates with the same major from different universities. For newly enrolled PhD students, it was common for them to compare themselves with previous college classmates. However, senior PhD students usually compare themselves with other fellow PhD students within the same institution and outside their institutions. For example, some students try to keep pace with previous students to attain the same quantity, or more, publications, while others felt worried about the need to compete with other PhD candidates/graduates in the job market.

*BF: “At the first 2–3 months after enrollment, when I was busy reading literature to propose a research topic, they (my former classmates studying for master’s degree in mainland China) have already started doing experiments and some even start writing paper, which brings me a great of stress.”*

*Laura: “Your various performances in PhD will be assessed, and that is, taking various courses, especially statistics classes. I am under great pressure to complete my project, especially when my colleagues around me work hard and excellent.”*

*Naruto: “My supervisor expects students to publish four or five papers in top journals within four years, and the former PhD students guided by my supervisor usually publish six papers before graduation. So, you must have a paper every seven or eight months, I feel bigger stress.”*

*Jianing: “You need to catch up with the speed of people competing with you, you need to publish more papers and faster than others, then you have chance to land that (job) position.”*

4. Difficulties shifting research directions/academic disciplines

Some participants mentioned that their PhD major is different from their major at the undergraduate/master’s levels. Exploring a new field can be puzzling and hard. In contrast to undergraduate or graduate education, in which the University provides a series of classes to help students grasp the core knowledge and skills, PhD education also expects students to possess critical thinking skills and original research ideas. For the students who change their major or research direction, setting aside what they were knowledgeable about and restarting to explore a new area were challenging.

*Lyman: “What I did here is cross-disciplinary research, my direction is different from other group members, and I am the only one doing experiments in the group. In the beginning, I didn’t have any experience of doing experiments, so basically all is to begin from nothing.”*

*Nie: “In fact, what I have learned before is not very relevant to my current major, so I have to learn a lot of things from scratch… so at first the stress is really huge.”*

*Kaylee: “It was a very stressful time at the beginning, I did policy research before, but now I have to add some models based on the disease progression, it is completely different from what I did before, now it is a little bit more clinical-focused, that is, you have to understand the clinical pathway of the disease.”*

5. Uncertainties about future career

PhD education could be an important steppingstone for one’s academic career. Compared with junior PhD students, senior PhD students are more concerned about their career development. Students who planned to work as research staff in colleges or institutions after graduation were also worried about the difficulties of attaining a decent job position. The increasingly stringent job recruitment criteria (i.e., in terms of the quantity and quality of publications) exacerbated the students’ stress.

*Jianing: “China’s current situation (in academia) is like a rising tide lifts all boats (“* *水* *涨船高” in Chinese, in our context it means the universities’ recruitment criteria for limited job positions become higher and stricter when more PhD graduates influx into the supply market), and then it’s involution (“* *内卷” in Chinese, in our context it means excessive competition in the academia, causing over qualification and evaluation pressure among PhD graduates).”*

*Naruto: “My supervisor hopes I can publish more articles, which is also want I intend to. Most PhD students want to find a teaching position in the future. If you don’t have enough publications, it will be difficult.”*

Pursuing a PhD is very time- and resource-intensive, with so much effort from the university, faculty and students. Even with those efforts, Some PhD students were still uncertain about their future career path; others did not want to find a job in academia.

*CY: “Actually I spend 6 years getting my PhD degree, my major is an extremely involution-intensive field (same as “* *内卷” in the above quotation of Jianing), and I’m fed up with this work. I don’t like research and I want to change to another job with higher pay.”*

*AX: “Pursuing a PhD narrows my career path; I’m anxious about graduation… I feel a little confused when I do my PhD, as I don’t know my future career direction. Compared with the time when I graduated with a master’s degree, the career road is limited now because I am older.”*

#### 3.2.2. Theme 2: Acculturative Stressors

In addition to academic stressors, the participants experienced difficulties adapting the life in Hong Kong. Their acculturative stressors included differences in the political environment, language barriers, difficulties living in Hong Kong, limited social interaction with others and local people’s discriminatory behaviors (Table 3). It is noteworthy that their acculturative stressors seemed to vary across different geographical regions of their hometowns. Participants from northern and western China experienced more difficulties in understanding and speaking Cantonese, in addition to adjusting to the local climate and food choices.

6. Differences in the political environment

Hong Kong is strongly influenced by both Western and Chinese cultures, potentially due to the prior occupancy by the British for 156 years. After the handover to China in 1997, Hong Kong maintained autonomy in the governing and economic systems from those of mainland China under the principle of “one country, two systems”. The participants perceived the differences in the political environment and beliefs between Hong Kong and China, which brought them a great deal of stress. We found that political issues were regarded as sensitive topics that mainland students usually avoided talking about. Such perceptions could affect how they select research supervisors, make friends and communicate with local people in daily life.

*Xie: “My major is politics and public administration, so when choosing my supervisor, I would avoid choosing people who are too ‘politically sensitive’ (which means one’s research may not be welcomed by the mainland academia for a political reason). If he/she is doing something about ethnic issues in Tibet or protesting, I would not consider it at all. I hope to work on some (politically) neutral topics.”*

*A: “I do have one good local friend. We get enrolled in the same year, and we get along well with each other. He holds a very friendly attitude towards the mainland (which means he does not support ‘Hong Kong is independent of China’), so it is the reason we have communication often and a close relationship.”*

To those participants, local people were highly involved in political activities. Many of the participants could not tolerate politically related violent behaviors (the participants mentioned some events that happened in 2019 during the social movement in Hong Kong). Those events brought them trauma-like psychological symptoms.

*Kaylee: “I’m not comfortable with this kind of political consciousness.”*

*Strawberry: “After the social movement happened in 2019, I was afraid of seeing local people, so I didn’t dare to adapt to local life.”*

*Max: “The difference exists in the value aspect, like last year’s (2019) social movement, this event has a significant (negative) impact on me… I don’t have any enthusiasm for being involved in this kind of political activity”.*

*Linwu: “I think those activities in 2019 bring a lot of psychological disturbances to me… hearing the gunshot and noises outside, I couldn’t sleep at that time, and I couldn’t stop browsing news on my mobile every other time to check on the news… I’m quite sure I was traumatized at that time”.*

7. Language barriers

As one of the participants (Willa) said, *“Language is an important part of adapting to the local environment.”* Cantonese is the first language in Hong Kong. Being a district dialect in mainland China, Cantonese is also the first language in southern China, particularly in the Guangdong province, which is geographically adjacent to Hong Kong. Compared to students from the Guangdong province or neighboring provinces, it is not surprising to note that participants from northern and western China experienced more difficulties in understanding and speaking Cantonese. One participant from the Fujian province (Lyman) said *“it (Cantonese) sounds a little bit like my hometown dialect.”* Even though participants from the Guangdong province did not experience difficulty in understanding and speaking in Cantonese, they also noted some subtle differences in the slang and expressions of the local people. It is also interesting to learn whether the participants perceived language as a barrier depending on how much they practically need to use it in daily life. Some students did not perceive a language barrier as they did not see a lot of need to interact with local people. Other students felt that their Cantonese proficiency improved as they stayed in Hong Kong longer.

*Jianing: “I pick up the southern accent very slowly because I have a northern accent. And when you talk to local people, sometimes they don’t understand, but they still prefer you to communicate in Cantonese. It’s like a small circle to me, you will feel being excluded from other people.”*

*Rita: “My major has changed, and I have to learn a lot of professional techniques, but there are no mainlanders in the laboratory, and daily communication with other lab mates could be a little hard.”*

*Stacey: “I think it is quite easy because I’m still in the regions with Cantonese as the native language, so speaking Cantonese is absolutely not a problem, but as Cici said: ‘Some expressions in daily communication are different (e.g., in mainland people use ‘* *摄像头’ to describe the computer-used camera whereas in Hong Kong people use ‘Cam’)’.”*

*Linwu: “We need to give students comments in Cantonese for our Teaching Assistantship work. I’m fine with Cantonese because I’ve been here for 4 years since 2016… However, it might be difficult for other new-enrolled doctoral students. Local undergraduate students would speak Cantonese directly to their mainland PhD TAs (Teaching Assistants), which makes those TAs quite confused.”*

8. Difficulties living in Hong Kong

The difficulties of living (accommodation, climate, and food) in Hong Kong also affect the participants’ adjustment to local life. Participants from northern China experienced more difficulties in adjusting to the climate and food in Hong Kong. To most of the participants, the accommodation was not spacious in Hong Kong. As they stayed longer in Hong Kong, they became used to the local residential environment and living conditions.

***Accommodation***

*Naruto: “Except the living area is really small, and there is nothing else I can’t adapt to.”*

*LC: “Housing is expensive but not worthwhile for its living condition… It is quite difficult to find a place where people live comfortably with satisfactory price levels and with satisfying roommates. Hard to find one ideal room.”*

***Climate***

*Max: “The ways of living are quite similar, such as the food and climate here. As I come from Fujian, also in southern China, I have no problem with climate or food.”*

*A: “At the beginning, the climate was not very suitable for me, because it’s dry in northern China but humid here. Then I got eczema in the first year, a little difficult to adjust, but not bad when I stayed here longer.”*

***Food***

*Qing: “When I first came to Hong Kong, I could not stand the food. I used to study in Sichuan before, so the food was stronger in taste. Then moved to Hong Kong, the food is all exceptionally bland and in Cantonese style (too much meat and extremely few vegetables, as said by Jianing).”*

*Willa: “I think it was because of my taste preference, but people from northern China won’t get used to the food here.”*

9. Limited social interactions with others

Many participants mentioned that their social interactions with people outside the academic settings were quite limited, regardless of this being with mainland students or local people. As one participant (PY) said, the *‘sense of boundary’* of people around made it hard to make friends. In most cases, mainland PhD students tended to develop social networks in the same workplace or same student hall. Physical proximity facilitated those students’ social interaction.

*Bean: “It is difficult to make friends after I came here. Especially hard to make close friends.”*

*PY: “Local people have a strong sense of boundaries with other people. In fact, it is not only among local students but also many mainland students in Hong Kong. I don’t know if they are assimilated into the environment of Hong Kong, if they change the way to interact with people after coming to Hong Kong, or if they behave in accordance with the characteristics of Hong Kong, probably the last reason.”*

The other factor affecting students’ social interaction was being occupied by their busy PhD study. Many participants mentioned that their academic workload occupied most of their time. To avoid unnecessary distractions, they were not motivated to make more local friends.

*Chen: “I wouldn’t say life here is difficult, but it’s definitely not easy. One is that I don’t have many friends after I came to Hong Kong, because my classmates I meet are members of my research group, and because I haven’t been involved in any student activities since I came here, therefore I don’t have many friends. That’s probably the main reason.”*

*Panda: “Honestly, I have little chance to communicate with local students. First, I spend most of my time on campus with classmates or friends. Secondly, the doctoral student network is dominated by mainland students here.”*

10. Local people’s discriminatory behaviors

A few participants shared their experiences of being discriminated against (both on and off campus). These participants experienced being treated unfairly because they spoke Mandarin or came from the mainland. Some local people treated them in an unfriendly way and felt that they were superior to the mainland people. The participants expressed their fear, discomfort and disappointment when talking about such experiences.

*Alice: “In 2019 (social movement) I don’t dare to speak Mandarin and I’m afraid there will be some people discriminating against me. Because there is a rumor that someone speaks Mandarin and then gets sprayed with paint on the subway.”*

*Willa: “I used to study at a university that has a lot of Hong Kong students in the mainland, at that time I can obviously feel their so-called superiority. And then after coming to Hong Kong, I always have such feelings. It’s not only about the language, and local people’s sense of superiority will make it more difficult to integrate into the society.”*

*Chen: “When I come to Hong Kong, I would like to make these local friends, but they can naturally distinguish you (mainland people) from local people. Just because of the different identity, they don’t understand you, put labels on you, and wear color class (“* *戴有色眼* *镜” in Chinese, in our context it means having prejudice and looking down at someone) to see you. These behaviors made me can’t communicate with them anymore.”*

## 4. Discussion

### 4.1. Theme 1: Academic Stressors

This study identified several important aspects of academic stressors among mainland Chinese PhD students in Hong Kong, including high expectations from the supervisors, emphasis on self-discipline as PhD students, peer comparison in academia, difficulties shifting research directions/academic disciplines and uncertainties about future careers. The findings were largely consistent with previous studies conducted in Western countries (e.g., the U.K., New Zealand) [18,19] and mainland China [67].

#### 4.1.1. High Expectations from the Supervisors

Similar to Wang and colleagues’ qualitative study among ten PhD students in mainland China [67], our participants reported distress about the lack of guidance on research. In both cases, supervisors were absent from their responsibilities, showed no interest or feedback on students’ research and did not give adequate support to the students. When proper equity and protection for students are in shortage in the supervisory relationship, it seems easier for those from East Asian universities (e.g., China, Korea, Japan) to accept this supervisor-centered, hierarchical relationship as they are more likely to endorse traditional Confucian values [68,69]. However, this phenomenon is unusual in European universities (e.g., German, Finland, Luxembourg) [70,71]. Culture may be a significant factor that affects the supervisory relationship in the academic environment.

There is also one special case—that is, when the student’s research is out of the supervisor’s expertise, such as conducting experiments or modeling—reflected by our participants; this is not a matter of responsibility, but rather, competence. We will discuss this situation in the following paragraph (difficulties shifting research directions/academic disciplines).

We highlight that supervisors’ expectations of academic publications also bring an additional stressor to the students. This finding was consistent with a prior study among 30 Chinese doctoral supervisors in different natural and social science disciplines [72]. Supervisors may pass their performance-appraisal-related stress to students. The standard for the quantity or quality of academic publications is likely to originate from the supervisor’s pressure to pursue the sustainability of their career path, such as attaining a higher level of academic position or obtaining a scientific foundation/grant. However, given that the participants did not specifically mention whether journal publications were prerequisites for their graduation, we could not rule out the possibility that those expectations for publication also come from the program requirements.

#### 4.1.2. Emphasis on Self-Discipline as PhD Students

Our finding was in line with previous studies conducted among Chinese international PhD students overseas (e.g., Australia, Finland, Malaysia) [47,73,74]. Meanwhile, our findings among junior PhD students and senior PhD candidates were also consistent with those reported by overseas PhD students. In particular, isolation brought about by owning a research topic and pursuing it alone [19], both intellectually and socially, poses challenges to students’ self-discipline, independence and time-management skills. Early-stage doctoral students tended to report pressure on proposing research questions and work-life balance [18], while late-stage PhD candidates were prone to report pressure on managing research progress, developing domain-specific expertise and meeting the requirements for graduation [20]. Moreover, as an individual characteristic, self-discipline is found to predict academic procrastination among international graduate students [75]. In this sense, it may be worthwhile to further explore the connection between students’ personal traits and their PhD experience.

#### 4.1.3. Peer Comparison in Academia

Peer comparison seems to be a commonly experienced stressor by our participants, especially for those majoring in science disciplines. It has been a trend that Chinese universities increasingly use the number of international publications as a major assessment and incentive measurement of their faculties’ academic performance, such as in the science and engineering disciplines [76]. As a result, the pressure for academic publication transfers to the recruitment process, which brings anxiety to the PhD candidates who aim to find a job in the academic field.

This does not only happen in China; Haven and colleagues [77] conducted a survey of all disciplinary fields and academic ranks among 1073 researchers in Amsterdam to explore their perceived publication pressure. The results showed that researchers in the humanities perceive greater publication stress than both biomedicine and the natural sciences. PhD students, especially PhD candidates, perceived a significant lack of resources to relieve publication stress. In our study, however, due to our study design, we could not compare the level of publication pressure; the limited number of participants from humanity disciplines also confine our findings.

#### 4.1.4. Difficulties Shifting Research Directions/Academic Disciplines

In contrast to prior findings, our participants highlighted the difficulties in changing discipline or research direction as another transition stressor. Many participants (primarily those in science majors) indicated that their research topic/direction did cater for interdisciplinary research needs. As indicated by Golde and colleagues [78], for students who need to conduct interdisciplinary research, finding a supportive advisor and a proper community, mastering knowledge and reconciling conflicting methodologies and overcoming fear were their major challenges. Those students were likely to experience stress due to their lack of qualifications and specialized training in the multiple disciplines needed for their research [79]. For early-stage doctoral students, changing discipline or research direction usually brings frustration and reduces the sense of belonging to a research group [80]. However, they tended to normalize that as a part of the “difficult” PhD journey.

#### 4.1.5. Uncertainties about Future Career

Consistent with PhD students in mainland China [67], many of our participants shared their plans to return to mainland China for seeking jobs as they worried about the limited job opportunities in the local market and the fierce competition with other scholars. Similar phenomena were also apparent in doctoral candidates, based on a scoping review of 26 studies conducted in Western countries [20].

It should be noted that most of our participants were year 2 and year 3 PhD students. They did not express urgent worries about their career path compared with senior PhD candidates. It is also noteworthy that all of our participants were not provided with any training/workshops specifically targeted to their career development. It would be important to explore whether those workshops could facilitate planning for their future career.

#### 4.1.6. Financial Concern Is Not a Stressor

In contrast to many PhD students studying in Western countries that deemed financial insecurity as the largest stressor [20], none of our participants reported financial pressures. A steady and secure financial income is a decisive factor for Chinese international doctoral students choosing to study abroad [81]. As Hong Kong’s publicly-funded universities offer PhD studentship stipends to their PhD students [82], it is possible that most of the full-time mainland PhD students have sufficient financial support for their research needs and living expenses.

### 4.2. Theme 2: Acculturative Stressors

This study identified several important aspects of acculturative stressors among mainland Chinese PhD students in Hong Kong, including differences in the political environment, language barriers, difficulties of living in Hong Kong, limited social interaction with others and local people’s discriminatory behaviors. Those stressors were largely consistent with those summarized by a previous review of international students’ difficulties studying abroad [21] and of local studies conducted among mainland students in Hong Kong universities [38,39,40].

#### 4.2.1. Language Barriers and Local People’s Discriminatory Behaviors

Among the stressors identified, language barriers and local people’s discriminatory behaviors are commonly experienced by mainland students in Hong Kong [38,39,40]. We further added evidence that whether participants have language barriers depends on where they come from and their practical needs. Specifically, compared with students from northern and western China, students from the Guangdong province or neighboring provinces (e.g., Fujian) have low difficulty listening and speaking Cantonese. Parallel to the cross-linguistic similarity in foreign language learning [83], the similarities of participants’ hometown dialect and Cantonese reduce their language obstacles. Additionally, whether the participants would perceive language as a barrier depends on the degree of their language proficiency and practical need in daily life. In an environment with the need to frequently interact with local people, such as discussions with supervisors and colleagues and fulfilling teaching assistance responsibilities, students with low Cantonese proficiency will have greater language difficulties.

Wang’s qualitative study showed that perceived discrimination was not a significant stressor among mainland Chinese undergraduates in a Hong Kong university because they were not disturbed in their daily lives [44]. Compared with the younger undergraduate participants in Wang’s study (*n* = 6), the PhD students in our study were expected to be more mature, autonomous, and politically concerned. They seemed to encounter more discrimination because of more interaction with local people and a greater likelihood of engaging in political debate or conflict. The tense political atmosphere during the social movement also exacerbated the discrimination towards mainland people to a higher level, which meant more possibilities of exposure to some local people’s discriminatory behaviors.

#### 4.2.2. Difficulties of Living in Hong Kong

In addition to the findings of Bhowmik’s study about mainland Chinese students’ difficulties living in Hong Kong (e.g., transport, food and accommodation) [40], we also revealed that subtle differences in climates due to the geographical locations of students’ hometowns are also relevant. For example, the climate in southern China is more humid than in northern China, and the cooking style in southern China is blander than in northern China. Such differences in daily living might also impact these students’ well-being. Therefore, future researchers and practitioners should consider the micro-level factors when examining academic sojourners’ adaptation to another place.

#### 4.2.3. Limited Social Interaction with Others

Limited social interaction with others was consistent with that reported by Yu and Zhang’s study among 54 mainland students from four Hong Kong universities [38] and Wang’s study among six mainland undergraduate students in a Hong Kong university [44]. In our study, we found that the interactional pattern among mainland students is affected by physical proximity. Similarly, as reported in Yu and Zhang’s study, the interactional pattern among mainland students is strongly influenced by collectivism in the mainland educational system, such as “four to six classmates living in the same dormitory, attending the same lectures and eating in the canteen together” [38]. In both studies, “language barrier” [38] and “skills of cross-cultural communication with Hong Kong local students were not adequate” [44] are reported as important reasons for limited social interaction, but were mentioned less frequently in our study. Compared with undergraduate and postgraduate students, PhD students are required to be more independent and more devoted to their academic research, which shapes students’ social interaction mode.

#### 4.2.4. Differences in the Political Environment

The difference in the political environment is an important acculturative stressor. As indicated in Yu and Zhang’s study [38], “Hong Kong is a highly politicalized city where opposing ideologies meet… One significant difference is the clashes of opposing political ideologies held by Hongkongers and Mainland students.” A typical example is that some Hong Kong people use ‘China’ to refer to ‘the mainland,’ which could be regarded as improper by mainland students because it seems to indicate that Hong Kong people do not think Hong Kong is a part of China. Living in a socialist society and influenced by its educational/media system for years, mainland students may feel uncomfortable about such sayings. As reflected by one participant in our study, holding an explicit political view (i.e., acknowledging Hong Kong is part of China) is the premise to establish friendship between mainland students and Hong Kong students.

We found that our participants tended to avoid being involved in sensitive political topics, both in academic research and social life. For those participants who planned to return to the mainland to seek academic positions, they would actively avoid choosing supervisors whose research was on politically conflicting/sensitive areas to prevent the potential negative effect this may have on their future career path. For those who experienced the impact of the social movement in 2019, they would choose to avoid political expression to protect themselves from potential physical harm and discrimination because of the terrifying rumor. Evidence showed that the social movement harms the quality of life and well-being of Hong Kong people [84], whereas academic sojourners (e.g., mainland students in Hong Kong in 2019), as witnesses of this social movement, were also inevitably affected. Our findings revealed the special acculturative stressor that can be created at a certain time; we call for more research to explore how social environment (e.g., social events, social policy) and individual factors (e.g., career path, personal experience) may affect one’s acculturation.

### 4.3. Implications

This study is one of the first studies to examine the adjustments among mainland Chinese PhD students studying in a different cultural setting, yet in the same country, from the perspective of the ELT Model. Our findings could help universities to identify the specific acculturative or academic needs among mainland Chinese PhD students in Hong Kong and provide recommendations for interventions to promote the improved academic and cultural adjustment of these students.

This study followed the dual transitions pointed out by the ELT model and enriched its application scenario to an intra-national context. On one hand, we remind future researchers to value both academic and acculturation transitions of international doctoral students when applying the ELT model in the cross-national context. On the other hand, our study adds new evidence to acculturation research that students who migrate for higher education within the same country also go through the process of acculturation, parallel to those who migrate between countries. Researchers should explore how the historical backgrounds of cities/states/provinces, the ways of living in hometowns (e.g., northern China vs. southern China) and social events/policy (e.g., the social movement in Hong Kong 2019) could bring subtle differences in people’s acculturative experiences, as well as the roles of culture/individual characteristics in affecting students’ academic experiences. Meanwhile, as China has long been the world’s biggest source of international students [85], many Chinese students go abroad for higher education. We also call for more research caring about the well-being of mainland Chinese PhD students overseas, adding more evidence and informing the practice.

To reduce acculturative stressors, cross-cultural training (CCT) could be provided by local universities for new PhD enrollers from the mainland. CCT refers to formal educational efforts to help elicit affective, behavioral and cognitive changes for improving cross-cultural adjustment and communication [86], which has been recommended for increasing students’ confidence and ability to adjust effectively in a cross-cultural setting, raising their awareness towards the norms and ways of living in the host society [87]. It may also be advised for universities to provide pre-departure online CCT to new mainland students [88]. Reliable information resources (e.g., online living guidance brochure, links for helping cultural adaption, brief introduction of services provided by universities) could be provided alongside the admission notice. After students’ arrival, CCT could be organized through seminars and workshops for new mainland students in Hong Kong [89]. In addition, having local and non-local students engage together in purposeful cross-cultural interaction may facilitate those students’ successful adjustment to the new learning and living environment [90]. Students are also encouraged to attend Cantonese learning classes to improve their language proficiency.

To reduce academic stressors, more support from the supervisors, the departments and the universities should be provided in the academic environment. For students who lack academic guidance on their research or suffer from a poor supervisory–student relationship (SSR), universities should establish formal regulations to guarantee academic interaction between supervisors and students. For example, detailed provisions on the frequency of meeting and communicating [91] and regular meetings can also cover content that improves students’ critical thinking and problem-solving methods [92]. Meanwhile, supervisors also need professional guidance for establishing good relationships with their students. Faculty may consider organizing regular cases-based workshops or professional development activities to support supervisors to build a good SSR [93]. Universities may also arrange term feedback from students to assess the quality of the SSR and the effectiveness of the supervisor’s guidance [94,95] and to assess whether an improvement or termination of the SSR should be made. In addition, experiences and lessons could also be summarized from mutual feedback for the future workshop’s use. Lastly, universities may use social network sites (e.g., Facebook, Instagram, WeChat) to promote mental health awareness [96], establish meaningful social connections [97] among PhD students and, eventually, improve their academic experience. Specifically, tips for the PhD journey, interaction zone and online emotional assistance could be embedded in existing social network platforms, the university’s exclusive applet or the official website to give social support to help students avoid a poor SSR, which could also enlarge students’ social interaction, help them find those with similar experience to themselves and enhance their psychological well-being.

For students who change their major or research direction, more emotional support, professional support [98] and timely feedback from departments and supervisors should be provided during their academic transition to help students establish confidence and skills when facing difficulties. For students who have problems establishing self-discipline and independence in their research, the department and supervisors may help them find the barriers, set goals of fostering self-discipline and create a climate that is supportive of a self-discipline emphasis [99]. For those experiencing uncertainties about their career paths, faculty may consider organizing job counseling workshops or seminars, inviting alumni from different career paths to share their experiences in a group format and providing informational support on job searching, interviewing skills or workplace behavior to current students [100].

### 4.4. Limitations

This study had limitations. Firstly, the use of purposive sampling as a non-probability sampling method may lead to a degree of selection bias in the recruitment [101]. In particular, the interviewees’ availability and willingness to participate are also important during recruitment [102]. Therefore, we may have missed some cases with other transition stressors or without any negative experiences. Considering this, the generalizability of the findings should not be exaggerated. Secondly, we observed that some participants avoided sharing their negative experiences or feelings in front of strangers due to social desirability bias [103]. Thirdly, we only included PhD students in our interviews; the inclusion of staff from universities, faculties and supervisors may provide a more comprehensive view and enhance the practical significance, for example, to explore the gaps between students’ needs and the existing services that have been provided. Further research may consider adopting the triangulation method to increase the credibility and validity of the research findings [104], using individual in-depth interviews or follow-up interviews [105]. Finally, it should be noted that this study was conducted during COVID-19 with different pandemic-control policies in place. A prior study has found that social distancing measures during the COVID-19 pandemic may delay students’ research progress [106], which may heighten students’ stress regarding their academic experiences. Those external factors might bring additional negative impacts on the academic/acculturative stressors experienced by the students.

## 5. Conclusions

Adjustment outcomes among mainland Chinese PhD students in Hong Kong should receive more empirical attention. Guided by the ELT Model, our findings identified specific academic stressors and acculturative stressors among mainland Chinese PhD students in Hong Kong. Theoretically, we extend the application scenario of the ELT model to the Asian context and within the same country (i.e., mainland China and Hong Kong); we also first adopt a comprehensive view of the education and life transitions of doctoral students in the cross-cultural setting.

For academic stressors, high expectations from the supervisor, emphasis on self-discipline as PhD students, peer comparison in academia and uncertainties about future careers were similar to those reported by previous studies among PhD students in mainland China and overseas. However, difficulties in changing discipline/research direction were identified as a special stressor in this study, which should not be ignored by the relevant scholars and practitioners. Moreover, financial concern was not a significant stressor in our study, which indicated that, as an important part of higher education, doctoral training systems vary in different countries/regions.

For acculturative stressors, the common stressors—namely language barriers, local people’s discriminatory behaviors, differences in the political environment, limited social interaction with others and difficulties of living in Hong Kong (e.g., accommodation)—basically overlap with those reported by previous local studies. Meanwhile, we added new evidence that the subtle differences (i.e., climate, food) caused by the different geographical regions also affect their adaptation. In addition, we gave a special spotlight to the social event, i.e., the social movement in Hong Kong in 2019; this typical example of the difference in the political environment poses challenges to students’ acculturation.

Practically, to better address those students’ academic and acculturative stressors, appropriate interventions (e.g., cross-cultural training), as well as support from the supervisors, the departments and the universities, should be provided. For future research, scholars should value both academic and acculturation transitions of international doctoral students in the cross-national education journey and their well-being. Researchers need to pay attention to exploring how the historical backgrounds of cities/states/provinces, the ways of living in hometowns and social events/policies could bring subtle differences in people’s acculturative experiences, as well as the roles of culture/individual characteristics in affecting students’ academic experiences.

## Figures and Tables

**Table 1:** Demographic characteristics of participants (*n* = 37).

No.	Alias	Gender	Major	PhD Study Year	Years in HK	Hometown
1	Hugh	Male	Arts	2	0.5–1	Anhui province, Southern China
2	Bean	Female	Social Science	4	≥3	Hebei province, Northern China
3	Xie	Female	Social Science	2	1–2	Jiangxi province, Southern China
4	Xing	Male	Engineering	2	1–2	Hubei province, Southern China
5	LC	Female	Business	3	2–3	Jiangsu province, Eastern China
6	Alice	Female	Business	3	≥3	Zhejiang province, Eastern China
7	Qing	Male	Science	2	2–3	Anhui province, Southern China
8	Patrick	Male	Science	≥5	≥3	Shaanxi province, Northern China
9	Jerney	Female	Engineering	4	≥3	Beijing, Northern China
10	Reedee	Male	Engineering	2	1–2	Tianjin, Northern China
11	Stacey	Female	Social Science	3	2–3	Guangdong province, Southern China
12	Lyman	Male	Engineering	2	1–2	Fujian province, East western China
13	Cici	Female	Social Science	1	0.5–1	Guangdong province, Southern China
14	XJ	Male	Medicine	2	≥3	Anhui province, Southern China
15	Alick	Male	Medicine	1	0.5–1	Shandong province, Northern China
16	Willa	Female	Law	2	2–3	Inner Mongolia Autonomous Region, Northern China
17	AD	Female	Medicine	2	≥	Shandong province, Northern China
18	John	Male	Science	3	2–3	Jiangxi province, Southern China
19	Jianing	Male	Engineering	2	0.5–1	Gansu province, Northwestern China
20	Nie	Female	Engineering	3	2–3	Jiangxi province, Southern China
21	Linwu	Female	Arts	3	≥3	Jiangsu province, Eastern China
22	Kaylee	Female	Medicine	3	2–3	Guangdong province, Southern China
23	PY	Male	Social Science	3	2–3	Sichuan province, Southern China
24	Rita	Female	Science	1	0.5–1	Shandong province, Northern China
25	BF	Male	Engineering	1	0.5–1	Beijing, Northern China
26	Strawberry	Female	Medicine	2	1–2	Beijing, Northern China
27	Max	Male	Science	4	≥3	Fujian province, Southern China
28	Chen	Male	Science	4	≥3	Jiangxi province, Southern China
29	CY	Male	Science	≥5	≥3	Jiangsu province, Southern China
30	Isaac	Male	Social Science	3	2–3	Shandong province, Northern China
31	Panda	Male	Business	≥5	≥3	Shanxi province, Northern China
32	Laura	Female	Medicine	1	≥3	Shandong province, Northern China
33	Carrot	Female	Social Science	2	1–2	Hubei province, Southern China
34	Meow	Female	Science	1	≥3	Jiangsu province, Southern China
35	A	Female	Science	3	≥3	Heilongjiang province, Northern China
36	Naruto	Male	Engineering	1	0.5–1	Shanxi province, Northern China
37	AX	Male	Engineering	3	≥3	Sichuan province, Southern China

**Table 2:** Theme: Academic stressors.

Sub-Theme	No. of Codes	No. of Participants Coded by This Sub-Theme
High expectations from the supervisors	21	13
Emphasis on self-discipline as PhD students	17	11
Peer comparison in academia	8	7
Difficulties shifting research directions/academic disciplines	5	5
Uncertainties about future career	5	5

**Table 3:** Theme: Acculturative stressors.

Sub-Theme	No. of Codes	No. of Participants Coded by This Sub-Theme
Differences in the political environment	27	23
Language barriers	17	11
Difficulties living in Hong Kong (i.e., Food, Climate, Accommodation)	14	14
Limited social interactions with others	8	8
Local people’s discriminatory behaviors	7	7

## Data Availability

The data presented in this study are available on reasonable request for academic use from the corresponding author after anonymization. The data are not publicly available to ensure the participants’ confidentiality.

## Supplementary Materials

The following supporting information can be downloaded at: https://www.mdpi.com/article/10.3390/ijerph20126078/s1, Table S1: Focus group interview questions; Table S2: Themes and subthemes from the focus group interview; Figure S1: Stressors of Mainland Chinese PhD students in Hong Kong during the Education and Life Transitions (ELT).

## References

[1] Pretorius L., Macaulay L., Cahusac de Caux B. (2019). Wellbeing in Doctoral Education: Intrapersonal Wellbeing and the Academic Mental Health Crisis. Wellbeing in Doctoral Education.

[2] Woolston C. (2022). Stress and uncertainty drag down graduate students’ satisfaction. Nature.

[3] Woolston C. (2017). Graduate survey: A love–hurt relationship. Nature.

[4] University of Westminster Nearly Half of PhD Students Consider Developing a Mental Health Problem ‘Normal’. https://www.westminster.ac.uk/news/nearly-half-of-phd-students-consider-developing-a-mental-health-problem-normal.

[5] Evans T.M., Bira L., Gastelum J.B., Weiss L.T., Vanderford N.L. (2018). Evidence for a mental health crisis in graduate education. Nat. Biotechnol..

[6] Barry K.M., Woods M., Warnecke E., Stirling C., Martin A. (2018). Psychological health of doctoral candidates, study-related challenges and perceived performance. High. Educ. Res. Dev..

[7] Levecque K., Anseel F., De Beuckelaer A., Van der Heyden J., Gisle L. (2017). Work organization and mental health problems in PhD students. Res. Policy.

[8] Guo L., Fan H., Xu Z., Li J., Chen T., Zhang Z., Yang K. (2021). Prevalence and changes in depressive symptoms among postgraduate students: A systematic review and meta-analysis from 1980 to 2020. Stress Health.

[9] Hazell C.M., Chapman L., Valeix S.F., Roberts P., Niven J.E., Berry C. (2020). Understanding the mental health of doctoral researchers: A mixed methods systematic review with meta-analysis and meta-synthesis. Syst. Rev..

[10] Nicholls H., Nicholls M., Tekin S., Lamb D., Billings J. (2022). The impact of working in academia on researchers’ mental health and well-being: A systematic review and qualitative meta-synthesis. PLoS ONE.

[11] Jindal-Snape D., Ingram R. (2013). Understanding and supporting triple transitions of international doctoral students: ELT and SuReCom models. J. Perspect. Appl. Acad. Pract..

[12] Schartner A., Young T.J. (2016). Towards an integrated conceptual model of international student adjustment and adaptation. Eur. J. High. Educ..

[13] Sarmiento A.V., Pérez M.V., Bustos C., Hidalgo J.P., del Solar J.I.V. (2019). Inclusion profile of theoretical frameworks on the study of sociocultural adaptation of international university students. Int. J. Intercult. Relat..

[14] Pho H., Schartner A. (2021). Social contact patterns of international students and their impact on academic adaptation. J. Multiling. Multicult. Dev..

[15] Najjar K.M. (2015). International Doctoral Students, Their Advising Relationships and Adaptation Experiences: A Qualitative Study. Ph.D. Thesis.

[16] Byrne E., Brugha R., McGarvey A. (2019). ‘A melting pot of cultures’–Challenges in social adaptation and interactions amongst international medical students. BMC Med. Educ..

[17] McGarvey A., Karivelil D., Byrne E. (2021). International Students’ Experience of Medical Training in an English-Speaking European Country. J. Stud. Int. Educ..

[18] Hockey J. (1994). New territory: Problems of adjusting to the first year of a social science PhD. Stud. High. Educ..

[19] Cornwall J., Mayland E.C., van der Meer J., Spronken-Smith R.A., Tustin C., Blyth P. (2018). Stressors in early-stage doctoral students. Stud. Contin. Educ..

[20] Mackie S.A., Bates G.W. (2018). Contribution of the doctoral education environment to PhD candidates’ mental health problems: A scoping review. High. Educ. Res. Dev..

[21] Smith R.A., Khawaja N.G. (2011). A review of the acculturation experiences of international students. Int. J. Intercult. Relat..

[22] Umaña-Taylor A.J., Alfaro E.C. (2009). Acculturative stress and adaptation. Handbook of US Latino Psychology: Developmental and Community-Based Perspectives.

[23] Berry J.W. (2006). Acculturative stress. Handbook of Multicultural Perspectives on Stress and Coping.

[24] Cox D. (1987). Migration/integration as a process: Welfare services for migrants: Can they be better planned. Int. Migr. Rev..

[25] Gordon L., Jindal-Snape D., Morrison J., Muldoon J., Needham G., Siebert S., Rees C. (2017). Multiple and multidimensional transitions from trainee to trained doctor: A qualitative longitudinal study in the UK. BMJ Open.

[26] Hellstén M. (2002). Students in transition: Needs and experiences of international students in Australia. Proceedings of the 16th Australian International Education Conference.

[27] Nasirudeen A., Josephine K.W.N., Adeline L.L.C., Seng L.L., Ling H.A. (2014). Acculturative stress among Asian international students in Singapore. J. Int. Stud..

[28] Steele K.D. (2008). Perceptions of Chinese International Students in Singapore: Adjustment Issues and Support. Ph.D. Thesis.

[29] Yusoff Y.M., Chelliah S. (2010). Adjustment in international students in Malaysian public university. Int. J. Innov. Manag. Technol..

[30] Lim C.-H. (2009). Acculturative stresses and adjustment elements of Chinese students’ studying in Korea. Korean J. Hum. Ecol..

[31] Guo Y., Li Y., Ito N. (2014). Exploring the predicted effect of social networking site use on perceived social capital and psychological well-being of Chinese international students in Japan. Cyberpsychol. Behav. Soc. Netw..

[32] Berry J.W. (2010). Mobility and acculturation. The Psychology of Global Mobility.

[33] Sun X., Chen M., Chan K.L. (2015). A meta-analysis of the impacts of internal migration on child health outcomes in China. BMC Public Health.

[34] Shi L., Chen W., Bouey J.H., Lin Y., Ling L. (2019). Impact of acculturation and psychological adjustment on mental health among migrant adolescents in Guangzhou, China: A cross-sectional questionnaire study. BMJ Open.

[35] Chan K.W. (2013). China: Internal migration. Encycl. Glob. Hum. Migr..

[36] Gui Y., Berry J.W., Zheng Y. (2012). Migrant worker acculturation in China. Int. J. Intercult. Relat..

[37] Hong Kong University Grants Committee Statistics of Hong Kong University Grants Committee. https://cdcf.ugc.edu.hk/cdcf/searchStatSiteReport.action.

[38] Yu B., Zhang K. (2016). ‘It’s more foreign than a foreign country’: Adaptation and experience of Mainland Chinese students in Hong Kong. Tert. Educ. Manag..

[39] Vyas L., Yu B. (2018). An investigation into the academic acculturation experiences of Mainland Chinese students in Hong Kong. High. Educ..

[40] Bhowmik M.K., Cheung R.Y.M., Hue M.T. (2018). Acculturative stress and coping strategies among Mainland Chinese university students in Hong Kong: A qualitative inquiry. Am. J. Orthopsychiatry.

[41] Poon A.Y.K. (2010). Language use, and language policy and planning in Hong Kong. Curr. Issues Lang. Plan..

[42] Zeng L.M. (2021). How much does it differ? How much does it matter? The research experience of Mainland Chinese and Hong Kong students in a Hong Kong University. Stud. High. Educ..

[43] Hoang A.P., Jordan L.P. (2019). Internationalisation and intersectionality in Hong Kong university student life: An exploratory study of social exclusion. Multicult. Educ. Rev..

[44] Wang P. (2017). Acculturation Stress and Acculturation Strategies: Mainland Chinese Undergraduate Students in the Education University of Hong Kong. Bachelor’s Thesis.

[45] Ye L.L. (2018). Setting the Scene for the Narratives to Follow. Intercultural Experience and Identity: Narratives of Chinese Doctoral Students in the UK.

[46] Hu Y., Zhao X., van Veen K. (2020). Unraveling the implicit challenges in fostering independence: Supervision of Chinese doctoral students at Dutch universities. Instr. Sci..

[47] Wang L., Räihä P. (2021). Academic acculturation of Chinese doctoral students in Finland. J. Humanit. Soc. Sci..

[48] Zhang Y.L. (2016). International students in transition: Voices of Chinese doctoral students in a US research university. J. Int. Stud..

[49] Rabiee F. (2004). Focus-group interview and data analysis. Proc. Nutr. Soc..

[50] Heggins W.J., Jackson J.F. (2003). Understanding the collegiate experience for Asian international students at a Midwestern research university. Coll. Stud. J..

[51] Mazzarol T., Soutar G.N. (2002). “Push-pull” factors influencing international student destination choice. Int. J. Educ. Manag..

[52] Huhn D., Huber J., Ippen F.M., Eckart W., Junne F., Zipfel S., Herzog W., Nikendei C. (2016). International medical students’ expectations and worries at the beginning of their medical education: A qualitative focus group study. BMC Med. Educ..

[53] Pan J.Y., Wong D.F.K., Chan C.L.W., Joubert L. (2008). Meaning of life as a protective factor of positive affect in acculturation: A resilience framework and a cross-cultural comparison. Int. J. Intercult. Relat..

[54] Central Policy Unit (2014). Mainland Students’ Adjustment in Hong Kong.

[55] Friedlander M.L., Ward L.G. (1984). Development and validation of the Supervisory Styles Inventory. J. Couns. Psychol..

[56] Millward L., Breakwell G.M., Hammond S., Fife-Schaw C. (1995). Focus Groups. Research Methods in Psychology.

[57] Ose S.O. (2016). Using Excel and Word to structure qualitative data. J. Appl. Soc. Sci..

[58] Ritchie J., Spencer L., Bryman A., Burgess R.G. (1994). Qualitative data analysis for applied policy research. Analysing Qualitative Data.

[59] Srivastava A., Thomson S.B. (2009). Framework analysis: A qualitative methodology for applied policy research. J. Adm. Gov..

[60] Church S.P., Dunn M., Prokopy L.S. (2019). Benefits to qualitative data quality with multiple coders: Two case studies in multi-coder data analysis. J. Rural Soc. Sci..

[61] Kurasaki K.S. (2000). Intercoder reliability for validating conclusions drawn from open-ended interview data. Field Methods.

[62] McAlister A., Lee D., Ehlert K., Kajfez R., Faber C., Kennedy M. Qualitative coding: An approach to assess inter-rater reliability. Proceedings of the 2017 ASEE Annual Conference & Exposition.

[63] McHugh M.L. (2012). Interrater reliability: The kappa statistic. Biochem. Med..

[64] Miles M.B., Huberman A.M. (1994). Qualitative data analysis: An expanded sourcebook. J. Environ. Psychol..

[65] Malterud K., Siersma V.D., Guassora A.D. (2016). Sample size in qualitative interview studies: Guided by information power. Qual. Health Res..

[66] Krueger R.A. (2014). Focus Groups: A Practical Guide for Applied Research.

[67] Wang X., Wang C., Wang J. (2019). Towards the contributing factors for stress confronting Chinese PhD students. Int. J. Qual. Stud. Health Well-Being.

[68] Zheng G., Shen W., Cai Y. (2018). Institutional logics of Chinese doctoral education system. High. Educ..

[69] Zhang Y.B., Lin M.-C., Nonaka A., Beom K. (2005). Harmony, hierarchy and conservatism: A cross-cultural comparison of Confucian values in China, Korea, Japan, and Taiwan. Commun. Res. Rep..

[70] Holmes P., Costa N., Lopes B., Stoicheva M., Byram M. (2019). The role of supervision in doctoral education–a transversal perspective. The Doctorate as Experience in Europe and Beyond: Supervision, Languages, Identities.

[71] Zheng G., Kivistö J., Shen W., Cai Y. (2019). Comparing doctoral education in China and Finland: An institutional logics perspective. Nordic-Chinese Intersections within Education.

[72] Wang X. (2022). Occupational Stress in Chinese Higher Education Institutions: A Case Study of Doctoral Supervisors. Int. J. Environ. Res. Public Health.

[73] Xu X., Sit H.H.W., Chen S., Xu X., Sit H.H.W., Chen S. (2020). Reflecting and Evaluating: Assessing the Doctoral Experience. The Eastern Train on the Western Track: An Australian Case of Chinese Doctoral Students’ Adaptation.

[74] Fei Z., Rasli A., Kowang T.O., Fei G.C., Koh H.P. (2023). Journey to the south: A case study of a Chinese PhD student in a Malaysian university. Int. J. Eval. Res. Educ..

[75] Alzangana K. (2017). Academic procrastination among international graduate students: The role of personality traits, the big-five personality trait taxonomy. Res. World.

[76] Tian M., Su Y., Ru X. (2016). Perish or publish in China: Pressures on young Chinese scholars to publish in internationally indexed journals. Publications.

[77] Haven T.L., Bouter L.M., Smulders Y.M., Tijdink J.K. (2019). Perceived publication pressure in Amsterdam: Survey of all disciplinary fields and academic ranks. PLoS ONE.

[78] Golde C.M., Gallagher H.A. (1999). The challenges of conducting interdisciplinary research in traditional doctoral programs. Ecosystems.

[79] Felt U., Igelsböck J., Schikowitz A., Völker T. (2013). Growing into what? The (un-) disciplined socialisation of early stage researchers in transdisciplinary research. High. Educ..

[80] Blackmore K.L., Nesbitt K.V. Identifying risks for cross-disciplinary higher degree research students. Proceedings of the Tenth Conference on Australasian Computing Education.

[81] Yang Y., Volet S., Mansfield C. (2018). Motivations and influences in Chinese international doctoral students’ decision for STEM study abroad. Educ. Stud..

[82] Parks S., Manville C., Stewart K., Lepetit L., Henham M.-L., Yang M., Chataway J. (2017). A Review of the Hong Kong Research Grants Council.

[83] Ringbom H. (2007). Cross-Linguistic Similarity in Foreign Language Learning.

[84] Shek D.T. (2020). Protests in Hong Kong (2019–2020): A perspective based on quality of life and well-being. Appl. Res. Qual. Life.

[85] Wen W., Hu D. (2019). The emergence of a regional education hub: Rationales of international students’ choice of China as the study destination. J. Stud. Int. Educ..

[86] Landis D., Bennett J., Bennett M. (2003). Handbook of Intercultural Training.

[87] Black J.S., Mendenhall M. (1990). Cross-cultural training effectiveness: A review and a theoretical framework for future research. Acad. Manag. Rev..

[88] Madden-Dent T., Laden R.M. (2016). Pre-departure cultural preparation for international students: Addressing adjustment needs before study abroad. Global Perspectives and Local Challenges Surrounding International Student Mobility.

[89] Tarique I., Caligiuri P. (2009). The role of cross-cultural absorptive capacity in the effectiveness of in-country cross-cultural training. Int. J. Train. Dev..

[90] Leung Y.W., Yu B. (2019). Making intercultural friends: How social and individual factors affect the Mainland-Hong Kong friendship network. Hong Kong Teach. Cent. J..

[91] Liang W., Liu S., Zhao C. (2021). Impact of student-supervisor relationship on postgraduate students’ subjective well-being: A study based on longitudinal data in China. High. Educ..

[92] Prazeres F. (2017). PhD supervisor-student relationship. J. Adv. Med. Educ. Prof..

[93] Eley A., Jennings R. (2005). Effective Postgraduate Supervision: Improving the Student/Supervisor Relationship.

[94] Davis D. (2019). Students’ Perceptions of Supervisory Qualities: What do Students want? What do they believe they receive?. Int. J. Dr. Stud..

[95] East M., Bitchener J., Basturkmen H. (2012). What constitutes effective feedback to postgraduate research students? The students’ perspective. J. Univ. Teach. Learn. Pract..

[96] Latha K., Meena K., Pravitha M., Dasgupta M., Chaturvedi S. (2020). Effective use of social media platforms for promotion of mental health awareness. J. Educ. Health Promot..

[97] Clark J.L., Algoe S.B., Green M.C. (2018). Social network sites and well-being: The role of social connection. Curr. Dir. Psychol. Sci..

[98] Jairam D., Kahl D.H. (2012). Navigating the doctoral experience: The role of social support in successful degree completion. Int. J. Dr. Stud..

[99] Rogus J.F. (1985). Promoting self-discipline: A comprehensive approach. Theory Into Pract..

[100] Arthur N., Nunes S. (2014). Should I stay or should I go home? Career guidance with international students. Handbook of Career Development.

[101] Sharma G. (2017). Pros and cons of different sampling techniques. Int. J. Appl. Res..

[102] Etikan I., Musa S.A., Alkassim R.S. (2016). Comparison of convenience sampling and purposive sampling. Am. J. Theor. Appl. Stat..

[103] Grimm P. (2010). Social desirability bias. Wiley Int. Encycl. Mark..

[104] Noble H., Heale R. (2019). Triangulation in research, with examples. Evid. Based Nurs..

[105] Gubrium J.F., Holstein J.A. (2001). Handbook of Interview Research: Context and Method.

[106] Donohue W.J., Lee A.S.-J., Simpson S., Vacek K. (2021). Impacts of the COVID-19 Pandemic on Doctoral Students’ Thesis/Dissertation Progress. Int. J. Dr. Stud..

