# Supplementary material for: “My Cross-Border PhD Journey”: A Qualitative Study on the Educational and Life Challenges of Mainland Chinese PhD Students in Hong Kong
Source: Int J Environ Res Public Health. 2023 Jun 7;20(12):6078. doi: 10.3390/ijerph20126078 (PMC10297838; doi:10.3390/ijerph20126078)
Supplement: Supplementary file 1 [file ijerph-20-06078-s001.zip › ijerph-2233316-supplementary.pdf]

International Journal of Environmental Research and Public Health

MDPI

Supplementary Table S1. Focus group interview questions.

Acculturation experience and stressors
- 'Do you think it easy or difficult living in Hong Kong? For example.'
- 'Do you feel any cultural difference between your hometown and Hong Kong, please describe that experience.'
- 'How do you feel about cultural differences in daily life? Is it stressful or not?'
Academic experience and stressors
- 'Do you think it easy or difficult in your Ph.D. study? For example.'
- 'How did you choose your Ph.D. supervisor? Is it different from your expectations?'
- 'How do you get along with your Ph.D. supervisor? Please briefly describe the mode of your interaction.'

Supplementary Table S2. Themes and subthemes from the focus group interview.

Themes	Sub-themes	No. of codes	No. of participants coded by this sub-theme
Academic stressors	High expectations from the supervisors	21	13
	Emphasis on self-discipline as Ph.D. students	17	11
	Peer comparison in academia	8	7
	Difficulties shifting research directions /academic disciplines	5	5
	Uncertainties about future career	5	5
Acculturative stressors	Differences in the political environment	27	23
	Language barriers	17	11
	Difficulties living in Hong Kong (i.e., Food, Climate, Accommodation)	14	14
	Limited social interactions with others	8	8
	Local people's discriminatory behaviors	7	7

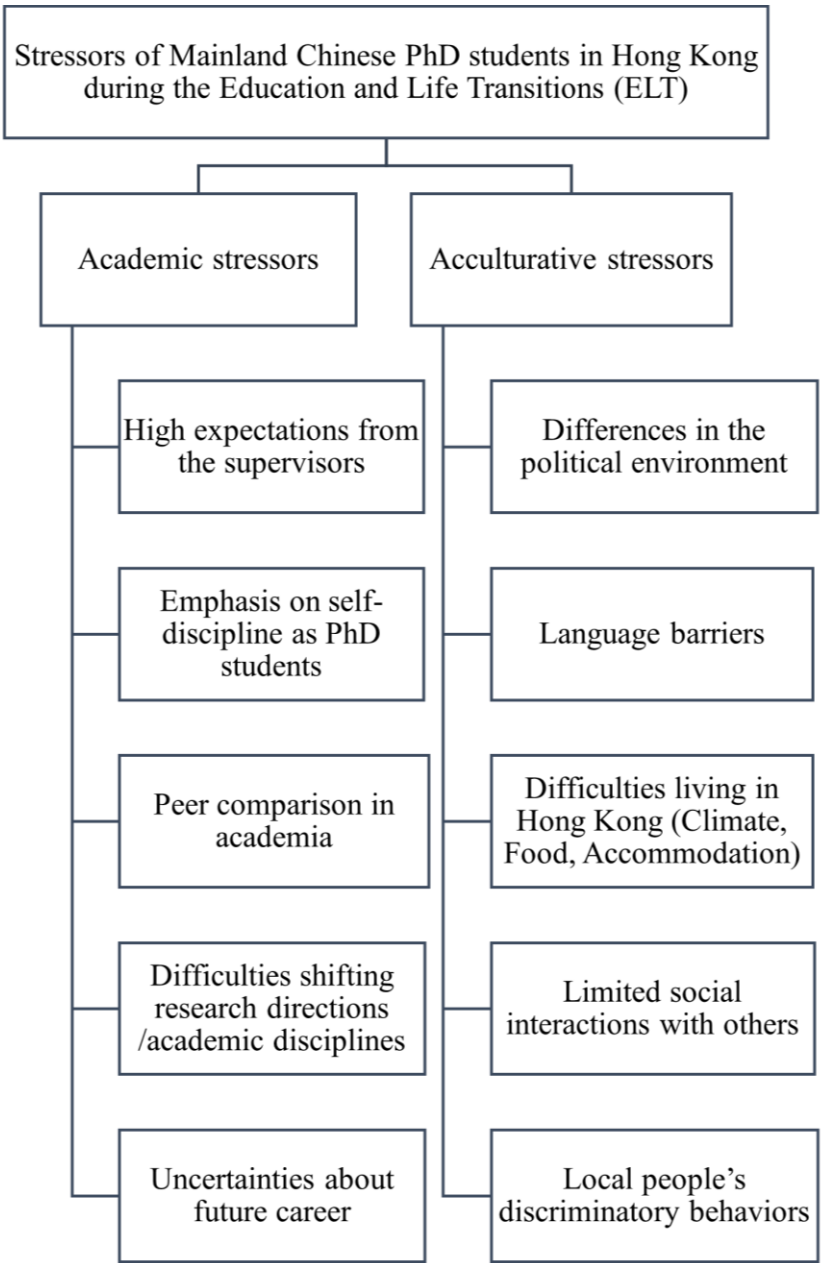

Supplementary Figure S1. Stressors of Mainland Chinese Ph.D. students in Hong Kong during the Education and Life Transitions (ELT).
